# Conduction System Pacing Improved Cardiac Functions, Myocardial Work and Functional Capacity in Heart Failure with Reduced Ejection Fraction and Right Bundle Branch Block

**DOI:** 10.3390/jcm15010232

**Published:** 2025-12-27

**Authors:** Anna Zsófia Tóth, László Nagy, Csaba Jenei, Arnold Péter Ráduly, Gábor Sándorfi, Krisztina Mária Szabó, Alexandra Kiss, László Tibor Nagy, Gergő István Szilágyi, Zoltán Csanádi

**Affiliations:** Division of Cardiology, Department of Cardiology, Faculty of Medicine, University of Debrecen, Móricz Zsigmond krt. 22., H-4032 Debrecen, Hungary; toth.anna@med.unideb.hu (A.Z.T.); csanadi.zoltan@med.unideb.hu (Z.C.)

**Keywords:** heart failure, cardiac resynchronization therapy, right bundle branch block, conduction system pacing, myocardial work and dyssynchrony

## Abstract

**Background/Objectives:** Conduction system pacing (CSP) is a potential alternative to biventricular pacing (BVP) in heart failure with reduced ejection fraction (HFrEF) and left bundle branch block (LBBB) or non-LBBB. Available data also suggest that unlike BVP, CSP may improve clinical outcome in patients with right bundle branch block (RBBB), although its effects on cardiac mechanics and energetics are ill-defined. Herein, we report on echocardiographic and clinical outcomes of CSP in this patient cohort. **Methods:** CSP either with His bundle pacing or LBB area pacing was attempted as a primary strategy in patients with RBBB, QRS duration ≥ 130 ms, LVEF < 35% and NYHA II-IV symptoms after optimized medical therapy for 6 months. Data on functional status, NT-proBNP and echocardiographic parameters were collected at baseline and 6 months after CSP. **Results:** CSP performed in 16 patients reduced QRS duration from 155.3 ± 12.8 ms to 130 ± 16.5 ms (*p* < 0.001), increased LVEF from 27 ± 7% to 33 ± 9% (*p* = 0.01), improved LV global longitudinal strain from −7 ± 3% to −10 ± 4% (*p* = 0.004) and improved LV peak strain dispersion from 126 ± 28 ms to 96 ± 23 ms (*p* = 0.004). Global myocardial work index increased from 582 ± 277 mmHg% to 840 ± 306 mmHg% (*p* = 0.003), as did global constructive work (900 ± 374 mmHg% to 1203 ± 393 mmHg%; *p* = 0.006) and global work efficiency (from 71 ± 7% to 77 ± 8%; *p* = 0.004). NYHA class (12.5% with NYHA II, 87.5% with NYHA III before vs. 25% with NYHA I, 50% with NYHA II and 25% with NYHA III at 6 months; *p* = 0.002) and 6 min walk distance (from 354 ± 88 m to 411 ± 95 m; *p* = 0.003) improved, while NT-proBNP decreased (from 4093 ± 7215 ng/L to 2087 ± 2872 ng/L, *p* = 0.003). **Conclusions:** CSP improved functional capacity and echocardiographic parameters related to cardiac functions and myocardial work in HFrEF patients with RBBB. Nevertheless, these results await further confirmation by large-scale, multi-center randomized trials.

## 1. Introduction

Cardiac resynchronization therapy (CRT) utilizing biventricular pacing (BVP) has been approved as an effective treatment for heart failure (HF) with reduced left ventricular (LV) ejection fraction (HFrEF), associated with significant intraventricular conduction delay. The most benefits after BVP can be expected in patients with a left bundle branch block (LBBB) pattern, while the improvement is less significant in the presence of a right bundle branch block (RBBB) morphology [1].

Conduction system pacing (CSP), including His bundle pacing (HBP) and left bundle branch area pacing (LBBAP), has recently emerged as an alternative to BVP to resynchronize the left ventricle (LV) in HFrEF patients with a bundle branch block (BBB) and a wide QRS complex. Unlike BVP, HBP as well as LBBAP were shown to achieve electrical resynchronization and improve clinical outcomes even in patients with RBBB and reduced LVEF [2,3]. LBBAP is a collective term for pacing strategies targeting the left bundle branch (LBB) region, including (1) direct stimulation of the main trunk of the LBB, referred to as LBB pacing (LBBP); (2) capture of the conduction system distal to the division of the main left bundle (left fascicular pacing); and (3) LV septal pacing (LVSP), capturing only the deep LV septal myocardium. In clinical practice, LBBAP has become the preferred method as opposed to HBP at many centers, considering its better long-term threshold stability and the technically less demanding implantation technique [4,5].

Reverse LV remodeling after resynchronization with BVP or with any of the CSP techniques mentioned above is assessed routinely by measuring the left ventricular ejection fraction (LVEF) using 2D echocardiography. However, recent data suggest that the evaluation of the global and regional myocardial deformation as well as myocardial work (MW) may provide a better and more comprehensive assessment of ventricular dyssynchrony and myocardial efficiency as well as further insights into the mechanism of improvement after CRT [6]. With the use of these novel and sensitive imaging techniques, the preservation of normal LV MW has been demonstrated with both HBP and LBBAP being performed for bradycardia indication in patients with a narrow QRS complex, while an improvement has been observed in those with HFrEF and LBBB [7]. However, no studies have yet investigated the effects of CSP on cardiac mechanics and energetics in HFrEF patients with RBBB.

We conducted this research to evaluate the efficacy of CSP on the LV myocardial performance, mechanical synchrony and MW in patients with advanced HF and RBBB ECG morphology.

## 2. Materials and Methods

### 2.1. Study Design and Patients’ Population

This was a single-center observational study based on a retrospective analysis of consecutive patients with CSP implantation between January 2021 and January 2024. HBP or LBBAP was attempted in patients with RBBB, QRS duration ≥ 130 ms, LVEF < 35% and NYHA class II to IVa HF symptoms despite guideline-directed medical treatment (GDMT) including beta-blockers, aldosterone antagonist, angiotensin-converting enzyme inhibitors (ACEIs)/angiotensin receptor blockers (ARBs)/angiotensin receptor-neprilysin inhibitors (ARNIs) and sodium-glucose co-transporter-2 (SGLT-2) inhibitors at least for 6 months. Patients with bradycardia indication for pacemaker therapy were excluded. The study protocol was approved by the ethics committee of the University of Debrecen (Registry number: BMEÜ/4388-1/2022/EKU), and the study complied with the Declaration of Helsinki.

### 2.2. CSP Implantation

Initially, HBP was the preferred method for CSP. Crossover to LBBAP was allowed in cases of failed HBP. However, in the last 6 patients, LBBAP became the preferred approach attempted first for CSP. HBP was performed using a Medtronic C315His sheath and SelectSecure 3830 lead (Medtronic, Minneapolis, MN, USA). His bundle (HB) region was identified by tricuspid valve angiography and then mapped with the pacing lead in unipolar fashion, recorded on an electrophysiology recording system (Schwarzer Cardiotek, Heilbronn, Germany). Once the HB potential was recorded, the lead was fixed by 4–6 clockwise rotations. The presence of HB capture, HB capture type (selective vs. non-selective HBP) and RBBB correction thresholds (detailed further in the Supplementary Materials) were assessed by decreasing the pacing output from 5V pulse amplitude and 0.4 ms pulse width as described earlier [8].

The methodology of LBBAP implantation has also been described [9]. After tricuspid valve angiography, the Medtronic C315His sheath and the SelectSecure 3830 lead were advanced 1.5–2 cm inside the right ventricle towards the apex. The target site for lead deployment was identified with unipolar pacing from the lead tip. Preferred QRS morphology was a ‘W’ pattern in V1 with discordant QRS complexes in lead II (positive or equiphasic) and III (negative or equiphasic). At this location, rapid rotation of the lead was applied, while fixation beats with RBBB morphology indicated the appropriate advancement. Rotations were then stopped, and the presence of LBB/fascicular potential, the unipolar paced QRS morphology, the myocardial current of injury and electric parameters were precisely evaluated. During LBBAP, unipolar stimulation was the preferred program setting. In case of inadequate RBBB pattern attenuation, bipolar stimulation was tested to overcome RBBB by anodal capture from the ring electrode [9].

### 2.3. Criteria of CSP Capture Types and Procedural Success

Selective (S-HBP) or non-selective HBP (NS-HBP) were determined based on previously published criteria. In general, in the case of S-HBP an isoelectric stimulus to QRS interval can be observed, while with NS-HBP a pseudo-delta wave following the stimulus indicates simultaneous myocardial activation in addition to the conductive tissue capture. In patients with bundle branch blocks (BBBs), both S-HBP and NS-HBP may result in partial/total BBB attenuation or the BBB may remain uncorrected (see the criterion in the Supplementary Materials) [10]. If a stable, reproducible HB capture with acceptable threshold (<3 V/0.4 ms) could not be achieved, or if the RBBB remained uncorrected, it was considered as a procedural failure.

LBBAP was confirmed when the unipolar paced QRS morphology in lead V1 demonstrated an RBBB pattern with one of the following criteria: (1) transition of the QRS morphology from either non-selective LBBP (NS-LBBP) to selective (S-LBBP) or NS-LBBP to LVSP during the unipolar threshold test by decreasing the pacing output from 5V pulse amplitude and 0.4 ms pulse width; or (2) the presence of LBB/fascicular potential with the interval of the potential to V6-R-Wave peak time (RWPT) in intrinsic rhythm equal to pacing stimulus to V6-RWPT. If none of the above criteria could be evaluated with the paced QRS complex showing Qr/QR morphology in lead V1, LVSP was confirmed. Failure of the procedure was defined as LBBAP with a high threshold, inability to penetrate the septum or failure to meet the predefined LBBAP criteria [3].

### 2.4. Echocardiographic Measurements

All echocardiographic measurements before and after CSP were carried out by the same cardiologist (C.J.) blinded to the clinical data of the patients to minimize any potential bias during interpretation of the echocardiographic results; however, intracardiac leads visible on echocardiographic images may be considered as a potential bias. Echocardiographic measurements including cardiac chamber quantification were performed according to the recommendations of the European Association of Cardiovascular Imaging [11]. Transthoracic echocardiographic images were recorded by using a Vivid E95 ultrasound system (GE Healthcare, Milwaukee, WI, USA), obtained for 3–5 consecutive beats for sinus rhythm, and at least 5 consecutive beats in the case of atrial fibrillation.

Left atrial diameters, LV and RV dimensions and volumes and tricuspid annulus plane excursion (TAPSE) were measured using 2D echocardiography, and LVEF was calculated by the modified biplane Simpson’s method. LV volumes were indexed on body surface area and expressed as mL/m^2^ [11]. Criteria for CSP response were defined as a ≥5% increase in the LVEF at 6 months follow-up, as used elsewhere [2,3].

Myocardial contractility and the intraventricular synchrony were further characterized by 2D speckle tracking echocardiography (2D-STE): LV global longitudinal strain (GLS) was considered as an advanced marker for the myocardial contractility, and the peak strain dispersion (PSD) implicated the LV mechanical dyssynchrony. Regional strain values were measured by 2D-STE at a frame rate of 60–100 frames/s by using images of the apical 4-, 3-, and 2-chamber views, as detailed elsewhere. GLS was calculated as an average of peak strains obtained from the 3 apical views. A bull’s-eye plot was created for peak GLS for each myocardial segment. PSD was defined as the standard deviation (SD) of the time-to-peak strain curves of different LV segments. Strain measurements were averaged from three cardiac cycles in sinus rhythm, or from 3–5 cycles in patients with atrial fibrillation [11,12].

LV MW was evaluated by the global myocardial work index (GWI), global constructive work (GCW), global wasted work (GWW) and global work efficiency (GWE), quantified by using LV pressure–strain loop (PSL) estimations paralleling non-invasive blood pressure (BP) measurements and LV GLS data. Non-invasive BP was recorded prior to the echocardiography at rest by averaging the results of three consecutive measurements; in patients with atrial fibrillation, BP average was obtained from 3–5 consecutive measurements. The area within each PSL implicated MW. The quantification of MW was carried out by automated function analysis on the software of the ultrasound system (GE Healthcare, Milwaukee, WI, USA). GWI (mmHg%) was considered as the amount of MW during systole, indicated by the area of PSLs from the mitral valve closure to mitral valve opening. GCW (mmHg%) represents the sum of positive work performed in systole (shortening) and negative work during isovolumetric relaxation (lengthening). GWW (mmHg%) was the sum of the negative work (lengthening) performed in systole and positive work (shortening) during isovolumetric relaxation. Finally, GWE was defined as the ratio of GCW to total work (GCW plus GWW), expressed as a percentage (%) [6].

### 2.5. Follow-Up

Patients were evaluated before the implantation and 6 months thereafter. Baseline ECG was evaluated with special care to differentiate typical and atypical RBBB patterns. Typical RBBB shows a wide S-wave in the lateral lead I and aVL, while an atypical RBBB pattern lacks an S-wave in lead I and aVL and is associated with left-axis deviation without or with PR prolongation [13]. Device interrogation, functional assessment (NYHA class evaluation and 6 min walk test), NT-proBNP level evaluation, detailed 2D echocardiography and 2D-STE were also performed. HF hospitalization and all-cause mortality data were also collected.

### 2.6. Statistical Analysis

GraphPad Prism 10 (GraphPad Software Inc., San Diego, CA, USA) was applied for statistical analysis. Continuous variables were depicted with the mean and SD; categorical variables were illustrated by counts and percentages. Chi-square distribution was used for evaluating the categorical variables. Paired comparisons were performed using a paired *t*-test if the data were normally distributed (estimated by D’Agostino and Pearson tests) and with the Wilcoxon test for nonparametric data. To account for multiple testing methods, *p*-values were adjusted using the Holm–Bonferroni method for the predefined outcome families.

## 3. Results

### 3.1. Patients’ Baseline Data

CSP implantation was attempted in 20 consecutive patients and concluded successfully with either HBP or LBBAP in 16, but deep septal penetration was not feasible in 4 patients. Baseline patient characteristics and comorbidities are depicted in Table 1. Briefly, male sex was overrepresented, and the underlying pathology was non-ischemic cardiomyopathy in two-thirds of the patients. The most common comorbidities were hypertension, diabetes mellitus, chronic kidney disease and atrial fibrillation.

Baseline ECG characteristics are summarized in Table 2. The mean QRS duration (QRSd) exceeded 150 ms, and only five patients had QRSd below 150 ms. The majority (12/16) of the patients had a typical RBBB pattern. Most patients also had atrioventricular (AV) conduction delay and/or a left anterior hemiblock pattern; however, none of them had advanced AV conduction disease requiring permanent cardiac pacing.

All patients were on optimized medical therapy: ACEI/ARBs and ARNI were used in 19% and 81%, respectively; beta-blockers in 100%; mineralocorticoid receptor antagonists in 93%; and SGLT-2 inhibitors in 63%, and no major changes in HF medication were introduced during the 6-month follow-up (Table S1). Additionally, no concurrent therapies including electrophysiological procedures, structural and coronary interventions were implemented during this period.

### 3.2. Periprocedural Data and Electrocardiographic Response

HBP, attempted primary in half of the study population, was successfully achieved in seven patients, and LBBAP was achieved in nine patients (LBBP: 6 and LVSP: 3). Periprocedural pneumothorax was observed in two patients, and pocket hematoma evacuation was needed in one patient. No other procedure-related complication was encountered. CSP lead revision due to the loss of His bundle capture was required in one patient and solved eventually with LBBAP (LVSP) shortly after the HBP lead implantation. Therefore, follow-up data were finally analyzed in 6 patients with HBP and 10 patients with LBBAP.

Table 3 summarizes the postoperative ECG features and the results of device interrogation and at 6-month follow-up. CSP resulted in a significant QRSd reduction indicated by delta QRS (25 ± 20 ms, *p* < 0.001). A complete elimination of RBBB was achieved by NS-HBP in all six patients, especially when higher pacing outputs were applied. In some patients, lower pacing output resulted in partial resolution of the RBBB pattern, while S-HBP resulted in no QRS correction in any patients. A marked decrease (20–40 ms) in QRSd with an incomplete attenuation of the RBBB pattern (reduction in R′ duration and amplitude) was observed in 6 out of 10 patients after LBBAP. QRSd increased in two patients after LBBAP. S-LBBP was intentionally not programmed, and pacing was delivered using NS-LBBP or LVSP. Anodal capture from the LBBAP lead during bipolar stimulation was used in 30% of patients during LBBAP.

### 3.3. Echocardiographic Response

Overall LVEF improved significantly from 27 ± 7% to 33 ± 9% (adjusted *p* = 0.01), and no change was observed in the RV function and diameter. Based on the echocardiographic criterion, 10/16 (62.5%) patients were classified as CSP responders, exhibiting almost a 10% increase in the LVEF. Improvement in LV function was in line with the echocardiographic changes indicating reverse LV remodeling. CSP resulted in a significant decrease in LV end-diastolic diameter (EDD, adjusted *p* = 0.01), end-systolic diameter (ESD, adjusted *p* = 0.001), as well as end-systolic volume (ESV, adjusted *p* = 0.02) and end-systolic volume index (ESVi, adjusted *p* = 0.02). After Holm–Bonferroni correction, no significant improvement was shown either in LV end-diastolic volume (EDV) and end-diastolic volume index (EDVi) or in stroke volume (SV) and stroke volume index (SVi; for further details, see Table 4).

### 3.4. LV Mechanical Outcomes

**Figure 1:**
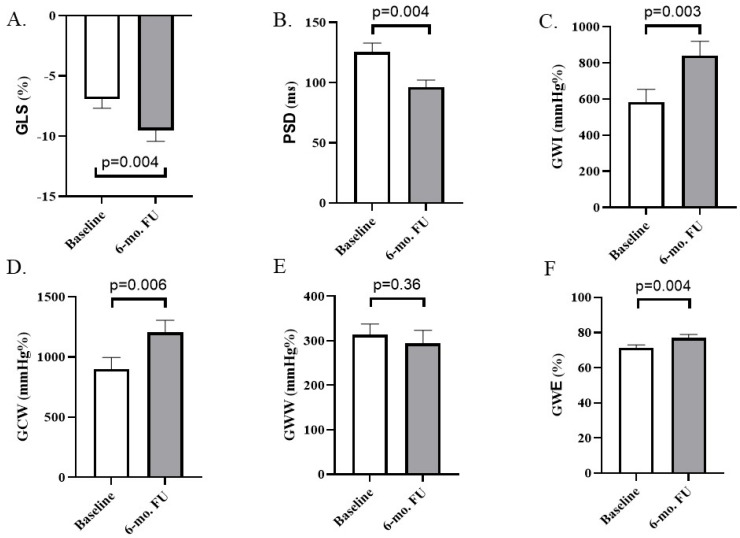
Changes in the LV mechanics associated with CSP including global LV longitudinal strain (**A**) and regional strain dispersion (**B**) and indices of the LV myocardial work (**C**–**F**). GLS: Global longitudinal strain, PSD: peak strain dispersion, GWI: global work index, GCW: global constructive work, GWW: global wasted work, GWE: global work efficiency. Values prior to implantation (baseline) and at 6-month follow-up are depicted, and adjusted *p*-values are shown.

LV mechanical performance was characterized by assessing GLS, myocardial dyssynchrony and MW parameters. LV GLS improved significantly from −7 ± 3% to −10 ± 4% (adjusted *p* = 0.004; Figure 1A). Myocardial dyssynchrony was characterized by PSD, which decreased from 126 ± 28 ms to 96 ± 23 ms (adjusted *p* = 0.004, Figure 1B). CSP resulted in significant improvement in GWI (840 ± 306 mmHg%) as compared with the baseline (582 ± 277 mmHg%; adjusted *p* = 0.003, Figure 1C). GCW also increased significantly from 900 ± 374 mmHg% to 1203 ± 393 mmHg% (adjusted *p* = 0.006, Figure 1D). Additionally, GWW demonstrated a numerical decrease (from 313 ± 94 mmHg% to 294 ± 114 mmHg%) at 6 months without reaching the level of statistical significance (adjusted *p* = 0.36, Figure 1E). GWE improved significantly from 71 ± 7% to 77 ± 8% (adjusted *p* = 0.004; Figure 1F).

### 3.5. Clinical Outcomes

**Figure 2:**
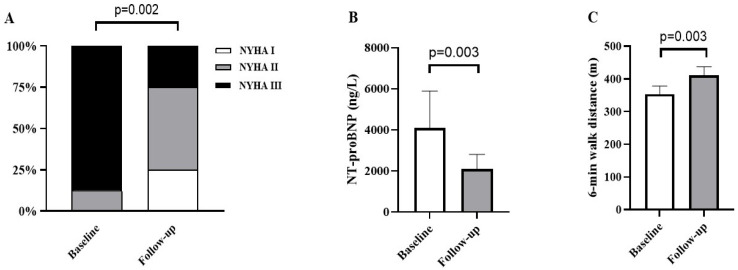
Effect of CSP on the clinical outcomes including changes in NYHA functional class (**A**), NT-proBNP level (**B**) and 6 min walk distance (**C**). Values prior to implantation (baseline) and at 6 months follow-up are depicted, and adjusted *p*-values are shown.

No HF hospitalization was required in any of the 16 patients during 6-month follow-up, and one patient was treated with electric cardioversion because of new-onset AF. An improvement of ≥1 NYHA functional class was demonstrated in 13 patients, while there was no change in 3 patients. Prior to the CSP, 12.5% of the patients were classified as NYHA II and 87.5% as NYHA III, while 25% were classified with NYHA I, 50% with NYHA II and 25% with NYHA III at 6 months (Figure 2A; adjusted *p* = 0.002). NT-proBNP level decreased significantly at the 6-month follow-up compared with the preoperative level (4093 ± 7215 ng/L at the baseline and 2087 ± 2082 ng/L at 6-month FU; adjusted *p* = 0.003; Figure 2B). Extremely elevated NT-proBNP (~35.000 ng/L) in one patient has disproportionately influenced variability. The 6 min walk distance increased significantly by an average of nearly 60 m (from 354 ± 88 m to 411 ± 95 m, adjusted *p* = 0.003; Figure 2C).

### 3.6. Subgroup Analysis

A subgroup analysis was performed between the patients’ cohorts treated with HBP versus with LBBAP (Table S2). Total procedure duration and fluoroscopy times, mean pacing threshold and R-wave sensing seemed to be more favorable with LBBAP as compared with HBP; however, after adjustment for multiple testing using the Holm–Bonferroni method, only the difference in the mean pacing threshold remained statistically significant (adjusted *p*-value = 0.019). No other between-group differences were found, either in the clinical outcomes or in the echocardiographic parameters; however, these comparisons were statistically underpowered owing to the limited sample size.

Additionally, baseline characteristics and perioperative data were comparable between CSP responders and non-responders; however, none of the above parameters reliably characterized these patient cohorts due to the small sample size within both groups.

## 4. Discussion

This was a single-center study on patients with HFrEF and RBBB ECG morphology undergoing CSP as a primary strategy for CRT. The main findings were as follows: (1) The significant reduction in QRSd after CSP was paralleled by signs of reverse cardiac remodeling and improved LV function. (2) CSP decreased the extent of LV dyssynchrony and improved LV MW. (3) CSP was associated with improved functional capacity, a lower NYHA functional class and reduced NT-proBNP levels.

Significant correlation has been demonstrated between the baseline ECG morphology and the long-term benefit after BVP. QRS width exceeding 150 ms and a typical LBBB pattern are considered the strongest predictor of resynchronization effect, while less benefit or even an increased mortality was reported in patients with RBBB [1,14]. A plausible explanation proposed for this finding could be that in patients with RBBB, the conduction delay is mainly present in the RV free wall and septum, leaving no room for significant improvement from LV free wall pre-excitation with BVP [15]. Additionally, recent studies suggest that RBBB is also associated with LV mechanical dyssynchrony in majority of the patients, although to a lesser degree than LBBB [16,17]. These findings are also supported by our data: significant LV mechanical dyssynchrony was indeed present in our patient cohort; however, no direct comparison with LBBB was available. In the present study, all patients fulfilled the guideline-based criteria (class IIa and b) for CRT implantation based on solely the QRSd and not the QRS morphology. However, considering the limited efficacy of BVP in RBBB [14] and emerging evidence supporting CSP as a more physiological alternative [18], CSP was selected here as the preferred resynchronization strategy over BVP. However, the present study is not intended to demonstrate superiority of CSP over BVP, but rather to characterize the effects of CSP in this specific patient cohort.

Correction or at least mitigation of the RBBB conduction delay pattern are considered signs of electrical resynchronization in patients with RBBB, although the mechanism is still not fully understood. In a recent report, permanent HBP applied as a primary strategy in patients with advanced HFrEF and RBBB was associated with significant narrowing in the QRSd and RBBB correction in 78% of patients (S-HBP in 34% and NS-HBP in 66%) [2]. In another study on patients with RBBB and preserved LVEF, NS-HBP resulted in a voltage-dependent attenuation of the RBBB pattern, which was not observed with S-HBP [19]. These results are in line with our observations: NS-HBP resulted in a voltage-dependent, complete QRS correction; however, no resolution of the RBBB pattern was associated during S-HBP. These findings may implicate the potential mechanism related to the modification of the RBBB pattern during HBP. The attenuation or correction of the RBBB pattern with NS-HBP but not with S-HBP may suggest a conduction block distal to the site of HBP and voltage-dependent capture of the right ventricular free wall myocardium or recruitment of parallel pathways resulting in pre-excitation of the area beyond the block. Even with no recruitment of RBB, the fusion between conduction over the His bundle (with RBBB pattern) and septal myocardial activation of the RV may still reduce the RV-LV activation delay [2,19,20].

HBP was performed in 6 of our patients, while LBBAP was performed in 10 of our patients. More than a 10 ms decrease in the QRSd with RBBB pattern attenuation was achieved in 60% of our patients who underwent successful LBBAP; however, QRSd increased in two patients. Both had apparent left-sided conduction system disease (left anterior hemiblock), and only LVSP was achieved. This phenomenon likely reflected LV septum activation with no effective recruitment of the conduction system leading to non-physiological ventricular activation. Comparable electrocardiographic response was demonstrated in the International LBBAP Collaborative Study [3]. Indeed, the magnitude of the QRS duration reduction observed in the present study is consistent with findings from recent reports [21,22]. This pattern of electric resynchronization was mainly achieved by either NS-LBBP or LVSP but less likely with S-LBBP, which might even be deleterious by inducing or preserving interventricular dyssynchrony and complete RBBB pattern. In contrast to S-LBBP, direct capture of the RV septal myocardium simultaneously with recruitment of the LBB conduction system observed both with NS-LBBP and LVSP might reduce the RV conduction delay. Nevertheless, LVSP was reported on worse clinical outcomes compared with LBBP, especially in those with reduced LVEF and wide QRS duration. While the magnitude of the interventricular dyssynchrony was comparable between these pacing strategies, NS-LBBP resulted in superior LV electrical and mechanical synchrony than LVSP [23]. These observations still warrant further validations in prospective randomized studies. Although CSP was associated with a significant reduction in LV mechanical dyssynchrony in our patient population, the limited sample size precluded robust subgroup analyses comparing HBP with LBBAP, as well as LVSP with LBBP. Further QRSd reduction could be achieved by anodal capture of the RV septum from the ring electrode with bipolar stimulation, which was present in 30% of our patients and in 48% of patients in the International LBBAP Collaborative study [24,25,26,27].

In addition to the RBBB, most of our patients had apparent left-sided conduction system disease (left anterior or posterior hemiblock) and prolonged AV conduction times (for further details see Table 2), which was similar to that reported in previous studies [2,3]. In the case of prolonged baseline PR interval, AV time optimization in addition to the correction of the RBBB pattern may contribute to the clinical improvement, as suggested by the findings of the HOPE-HF and a sub-analysis of the MADIT-CRT study [14,28,29]. Atypical RBBB, also referred to as masked LBBB, was found in 25% of our patients. The size of our patient cohort did not allow us to assess the impact of the above ECG features; however, the International LBBAP Collaborative Study found neither the baseline left-sided conduction delay nor the RBBB pattern normalization predictive for clinical response after LBBAP. Reduction in the paced QRS duration as compared with the baseline was the only independent predictor of the echocardiographic response [3].

Global and regional myocardial deformation and myocardial work (MW) was used to gain further insight into the mechanism of improvement after CRT, providing a loading-independent evaluation of myocardial performance and myocardial efficiency. Electro-mechanical synchronization of the chambers was reported to improve cardiac efficiency and energetics translating into an improvement in myocardial function and better outcomes, and both was achieved in our patient cohort [30]. A significant correlation was described between the global LV MW and the long-term prognosis in patients with various cardiac diseases [31]. Furthermore, both HBP and LBBAP have been associated with favorable changes in the cardiac energetics and MW parameters implemented in a wide range of pacing indications [7,32]. In our patient cohort, CSP increased GWI, GCW and GWE, which might be hypothesized as a potential link between improved mechanical synchrony and better echocardiographic and clinical outcomes. Nevertheless, the absence of any control group (e.g., optimal medical therapy alone or conventional BVP) represents a significant limitation in the interpretation of these findings, and hence, clear causality cannot be inferred.

## 5. Conclusions

To the best of our knowledge, this is the first study providing a detailed analysis of the mechanical synchrony and myocardial work after CSP in HFrEF patients with RBBB. We demonstrated that CSP with either HBP or LBBAP decreased LV mechanical dyssynchrony, which might lead to improved myocardial work and efficiency. Furthermore, CSP resulted in reverse cardiac remodeling and better clinical outcomes, which may support its use for cardiac resynchronization in this patient cohort. However, these data should be viewed as hypothesis-generating until being validated by a randomized trial including an adequate number of patients.

## 6. Limitations

This was a single-center, observational, non-randomized study including a limited number of patients. Such a limited sample may restrict statistical power, making it particularly challenging to perform subgroup analyses comparing HBP and LBBAP. In line with this, no baseline and postoperative parameters reliably discriminated CSP responders from non-responders due to the limited sample size. Another limitation is the lack of any control group or a direct comparison of CSP with the conventional BVP. Based on these limitations, any extrapolation from our data should be interpreted with caution before being validated in a prospective, randomized trial including a sufficient number of patients receiving optimized medical therapy alone or conventional BVP. Changes in HF symptoms, functional status and echocardiographic outcomes were used as the primary endpoint in our research with no results on long-term survival. Spontaneous fluctuations in HF status and delayed effects of medical therapy optimization cannot be fully excluded.

Although patients were treated with stable, optimized GDMT, medication adherence and other unmeasured concurrent interventions could not be formally assessed and may have contributed to the observed outcomes. Despite standardized, blinded image acquisition and analysis, inter- and intra-observer variability of advanced echocardiographic parameters (GLS, PSD, myocardial work) were not formally evaluated.

## Figures and Tables

**Table 1:** Patient’s baseline data and comorbidities. BMI: body mass index, BSA: body surface area, GFR: glomerular filtration rate, TIA: transitional ischemic attack.

Age, years, mean ± SD	63.9 ± 10.7
Male gender, *n* (%)	15 (93.8)
BMI, kg/m^2^, mean ± SD	27.3 ± 5.7
BSA, m^2^, mean ± SD	1.95 ± 0.23
Creatinine, μmol/L, mean ± SD	107.7 ± 47.5
GFR, mL/min	63 ± 17
Hemoglobin, g/L, mean ± SD	138.8 ± 20.9
Diabetes Mellitus, *n* (%)	7 (43.8)
Hypertension, *n* (%)	7 (43.8)
Chronic kidney disease, *n* (%)	5 (31.3)
Ischemic cardiomyopathy, *n* (%)	6 (37.5)
Atrial fibrillation, *n* (%)	4 (25.0)
TIA/stroke, *n* (%)	0 (0)

**Table 2:** Baseline ECG characteristics of the patients’ population. RBBB: right bundle branch block, AV: atrioventricular.

Baseline QRS, ms, mean ± SD	155.3 ± 12.8
Baseline PR-interval, ms, mean ± SD	223.1 ± 33
Isolated RBBB, *n* (%)	4 (25)
Typical RBBB, *n* (%)	12 (75)
Atypical RBBB, *n* (%)	4 (25)
Bifascicular block, *n* (%)	8 (50)
Trifascicular block, *n* (%)	4 (25)
High degree AV block, *n* (%)	0 (0)

**Table 3:** Postop. ECG characteristics and 6 mo. interrogation data of the implantable cardiac device. Delta QRS was calculated as the difference value between the preoperative and postoperative QRS duration; V6-RWPT: R-wave peak time in lead V6 during LVSP and LBBP, CSP: conduction system pacing, AT/AF: atrial tachycardia/atrial fibrillation, Vp: ventricular pacing.

Postoperative QRS duration, ms, mean ± SD	129.9 ± 16.5
Delta QRS, ms, mean ± SD	25.4 ± 19.9
V6-RWPT, ms, mean ± SD	82.1 ± 8
CSP-lead impedance, ꭥ, mean ± SD	622.3 ± 163
R-Wave amplitude, mV, mean ± SD	8.8 ± 5.9
CSP-lead threshold, V at 0.4 ms pulse width, mean ± SD	1.6 ± 1.1
AT/AF burden, %, mean ± SD	6.57 ± 24.9
Vp burden, %, mean ± SD	97.8 ± 3.1

**Table 4:** RV and LV dimensions and LV stroke volumes at baseline and at 6-month follow-up (FU). LV volumes were indexed at BSA and expressed by mL/m^2^. Values are depicted as the mean ± SD. RVD1: right ventricular internal diameter at the tricuspid valve, TAPSE: tricuspid annulus plain excursion, EF: ejection fraction, LV: left ventricle, ESD: end-systolic diameter, EDD: end-diastolic diameter, ESV: end-systolic volume, ESVi: ESV index, EDV: end-diastolic volume, EDVi: EDV index, SV: stroke volume, SVi: SV index. Statistical significance is highlighted by bold.

	Baseline	6-Month FU	*p*-Value	Adjusted *p*-Value
RVD1, mm, mean ± SD	42.6 ± 5.9	41.8 ± 7.7	0.52	1
TAPSE, mm, mean ± SD	17.31 ± 3.2	17.44 3.14	0.88	1
LV EF, %, mean ± SD	27.4 ± 6.9	33.3 ± 8.5	0.001	**0.01**
LV ESD, mm, mean ± SD	55.4 ± 11.4	51.0 ± 11.7	<0.0001	**0.001**
LV EDD, mm, mean ± SD	67.3 ± 9.2	64.5 ± 9.6	0.001	**0.01**
LV ESV, mL, mean ± SD	146.2 ± 65.7	124.6 ± 53.7	0.003	**0.02**
LV ESVi, mL/m^2^, mean ± SD	74.4 ± 31.3	63.5 ± 25.4	0.003	**0.02**
LV EDV, mL, mean ± SD	194.9 ± 76.3	180.7 ± 59.3	0.04	0.16
LV EDVi, mL/m^2^, mean ± SD	99.5 ± 20.7	92.4 ± 30	0.04	0.16
LV SV, mL, mean ± SD	48.8 ± 19.3	56.1 ± 15.5	0.02	0.12
LV SVi, mL/m^2^, mean ± SD	25.1 ± 10.4	28.8 ± 8.9	0.02	0.12

## Data Availability

Anonymized data are available upon reasonable request. (Debrecen University Hospital IT System: UD Med).

## Supplementary Materials

The following supporting information can be downloaded at: https://www.mdpi.com/article/10.3390/jcm15010232/s1. Table S1. Guideline-directed medical therapy of the patient cohort. Table S2. Comparison between HBP (6 patients) and LBBAP (10 patients).

## Abbreviations

The following abbreviations are used in this manuscript:

2D-STE	Two-dimensional speckle tracking echocardiography
ACEI	Angiotensin-converting enzyme inhibitor
ARB	Angiotensin receptor blocker
ARNI	Angiotensin receptor–neprilysin inhibitor
BBB	Bundle branch block
BP	Blood pressure
BVP	Biventricular pacing
CRT	Cardiac resynchronization therapy
CSP	Conduction system pacing
EDD	End-diastolic diameter
EDV	End-diastolic volume
EDVi	End-diastolic volume index
ESD	End-systolic diameter
ESV	End-systolic volume
ESV	End-systolic volume index
GCW	Global constructive work
GDMT	Guideline-directed medical treatment
GLS	Global longitudinal strain
GWE	Global work efficiency
GWI	Global work index
GWW	Global wasted work
HB	His bundle
HF	Heart failure
HFrEF	HF with reduced ejection fraction
LBBAP	Left bundle branch area pacing
LBBB	Left bundle branch block
LFP	Left fascicular pacing
LV	Left ventricle
LVEF	Left ventricular ejection fraction
LVSP	Left ventricular septal pacing
ms	Milliseconds
MW	Myocardial work
NS-HBP	Non-selective His bundle pacing
NS-LBBP	Non-selective left bundle branch pacing
PSD	Peak strain dispersion
PSL	Pressure–strain loop
QRSd	QRS duration
RBBB	Right bundle branch block
RV	Right ventricle
SGLT-2	Sodium-glucose co-transporter 2
S-HBP	Selective His bundle pacing
S-LBBP	Selective left bundle branch pacing
SV	Stroke volume
SVi	Stroke volume index
TAPSE	Tricuspid annular plane systolic excursion
