# Supplementary material for: Conduction System Pacing Improved Cardiac Functions, Myocardial Work and Functional Capacity in Heart Failure with Reduced Ejection Fraction and Right Bundle Branch Block
Source: J Clin Med. 2025 Dec 27;15(1):232. doi: 10.3390/jcm15010232 (PMC12786765; doi:10.3390/jcm15010232)
Supplement: Supplementary file 1 [file jcm-15-00232-s001.zip › jcm-4051218-supplementary.pdf]

Journal of
Clinical Medicine

MDPI

Supplementary Materials

According to the capture type (S-HBP or NS-HBP) and the presence or absence of conduction delay correction, 4 different phenomena can be observed in patients with RBBB during HBP.

1. **S-HBP with correction of BBB:** The interval between the pacing artefact and onset of the QRS complex (S–QRS) ≤ the interval between the His-potential and onset of the QRS complex (H-QRS) with an isoelectric interval between stimulus to onset of QRS. The paced QRSd will be narrower than the native QRS and the BBB may be completely normalized or partially corrected.
2. **S-HBP without correction of BBB:** S-QRS interval is usually equal to the H-QRS interval, the paced QRSd will be equal to the native QRS.
3. **NS-HBP with correction of BBB:** S-QRS interval is 0 or less than H-QRS interval due to the absence of isoelectric interval attributable to the pseudo-delta wave reflecting simultaneous myocardial activation. Paced QRS duration will usually be less than the native QRS and the BBB may be completely normalized or partially corrected.
4. **NS-HBP without correction of BBB:** S-QRS interval is 0 or less than H-QRS depending on the amount of pre-excitation. The paced QRSd is usually longer than the native QRSd. However, in patients with RBBB, NS-HBP may significantly narrow the QRS even in the absence of BBB correction due to fusion of left bundle activation with early anteroseptal RV activation.

Table S1. Guideline-directed medical therapy of the patient cohort; ARNI: angiotensine-receptor neprilyzine inhibitor, ACEI: angiotensine receptor inhibitor, ARB: angiotensine receptor blocker, MRA: mineralocorticoide receptor antagonist, SGLT2-I: sodium-glucose transporter-2 Inhibitor.

ACEI/ARB, n (%)	3 (18.8)
ARNI, n (%)	13 (81.3)
Beta blocker, n (%)	16 (100)
MRA, n (%)	15 (93.8)
SGLT2-I, n (%)	10 (63)
Diuretics, n (%)	16 (100)

Table S2. Comparison between HBP (6 patients) and LBBAP (10 patients). Values were depicted as mean ± SD. Delta values indicated the difference between baseline and postoperative (6-mo. follow-up) data.

	HBP	LBBAP	p-value	Adjusted p-value
CSP lead sensing, mV, mean ± SD	3.7 ± 1.6	11.2 ± 5.7	0.008	0.144
CSP lead treshold, V, at 0.4 ms pulse width, mean ± SD	2.8 ± 1.2	1.2 ± 0.4	**0.001**	**0.019**
Procedure time, min, mean ± SD	116 ± 31.3	89.7 ± 17.9	0.049	0.784
Scialoscopy time, min, mean ± SD	81.8 ± 12.6	20.1 ± 6	0.02	0.34
Delta QRS, ms, mean ± SD	38.3 ± 17.1	32.6 ± 17.1	0.46	1.0
Delta NT-proBNP, ng/l, mean ± SD	-1263.3 ± 1551.7	-2452.8 ± 5803.1	0.64	1.0
Delta 6-min walk distance, m, mean ± SD	77.4	44	0.3	1.0
Delta LVEF, %, mean ± SD	6 ± 5.6	5.7 ± 5	0.91	1.0
Delta SVi, ml/m², mean ± SD	5.3 ± 2.5	3.6 ± 5.8	0.55	1.0
Delta ESVi, ml/m², mean ± SD	-13.4 ± 12.8	-8.7 ± 0.4	0.42	1.0
Delta EDVi, ml/m², mean ± SD	-8.1 ± 13	-5.7 ± 10.4	0.71	1.0
Delta ESD, mm, mean ± SD	-6 ± 3.2	-3.4 ± 2.3	0.08	1.0
Delta EDD, mm, mean ± SD	-3 ± 2.8	-2.7 ± 2.8	0.84	1.0
Delta PSD, ms, mean ± SD	-42.5 ± 26	-22.6 ± 29.2	0.22	1.0
Delta GLS, %, mean ± SD	-3.14 ± 2	-2.34 ± 2.7	0.57	1.0
Delta GWI, mmHg%, mean ± SD	363.4 ± 216	204.6 ± 219.5	0.21	1.0
Delta GCW, mmHg%, mean ± SD	385.6 ± 187.3	261.8 ± 202.8	0.27	1.0
Delta GWW, mmHg%, mean ± SD	-37.8 ± 91	-10.1 ± 75.7	0.54	1.0
Delta GWE,%, mean ± SD	8.8 ± 5.3	4 ± 4.6	0.1	1.0
